# Development of a digital star‐shot analysis system for comparing radiation and imaging isocenters of proton treatment machine

**DOI:** 10.1002/acm2.14320

**Published:** 2024-03-07

**Authors:** Ji Hye Han, Kwanghyun Jo

**Affiliations:** ^1^ Department of Physics Ewha Womans University Seoul South Korea; ^2^ Department of Radiation Oncology Samsung Medical Center Seoul South Korea

**Keywords:** linear accelerator, plastic scintillator, proton therapy, quality assurance, radiation isocenter, Raspberry Pi

## Abstract

**Purpose:**

To directly compare the radiation and imaging isocenters of a proton treatment machine, we developed and evaluated a real‐time radiation isocenter verification system.

**Methods:**

The system consists of a plastic scintillator (PI‐200, Mitsubishi Chemical Corporation, Tokyo, Japan), an acrylic phantom, a steel ball on the detachable plate, Raspberry Pi 4 (Raspberry Pi Foundation, London, UK) with camera module, and analysis software implemented through a Python‐based graphical user interface (GUI). After kV imaging alignment of the steel ball, the imaging isocenter defined as the position of the steel ball was extracted from the optical image. The proton star‐shot was obtained by optical camera because the scintillator converted proton beam into visible light. Then the software computed both the minimum circle radius and the radiation isocenter position from the star‐shot. And the deviation between the imaging isocenter and radiation isocenter was calculated. We compared our results with measurements obtained by Gafchromic EBT3 film (Ashland, NJ, USA).

**Results:**

The minimum circle radii were averaged 0.29 and 0.41 mm while the position deviations from the radiation isocenter to the laser marker were averaged 0.99 and 1.07 mm, for our system and EBT3 film, respectively. Furthermore, the average position difference between the radiation isocenter and imaging isocenter was 0.27 mm for our system. Our system reduced analysis time by 10 min.

**Conclusions:**

Our system provided automated star‐shot analysis with sufficient accuracy, and it is cost‐effective alternative to conventional film‐based method for radiation isocenter verification.

## 1 INTRODUCTION

Modern radiotherapy using state‐of‐the‐art technologies, such as intensity or volumetric modulation, can achieve a high level of conformity. As a result, the treatment outcome is very sensitive to small errors in anatomy matching or in beam delivery. Additionally, the expansion of hypo‐fractionated radiotherapy, which uses reduced number of fractions, limits the ability to compensate for errors. Therefore, quality assurance (QA) of the treatment machine becomes more important for accurate beam delivery. Several QA protocols, recommendations, and guidelines have been developed to address this issue.^1^, ^2^

One of the important procedures is the measurement of the position of the radiation isocenter, because any deviations could lead to systematic errors in delivering the radiation beam. A star‐shot analysis is a common method to verify the radiation isocenter position, narrow beams are irradiated at various angles to obtain a star‐shaped beam pattern on a radiosensitive film. Each narrow beam is transformed into a line, and intersection points of lines are encompassed by a small circle.^3^, ^4^ The radius of this circle represents the minimum circle radius, and its center corresponds to the radiation isocenter.^5^

The conventional radiation isocenter measurement method, film‐based star‐shot analysis, is well‐known and accurate. However, it cannot directly compare the radiation isocenter to the imaging isocenter. Since patients are positioned by using the imaging device, the beam delivery accuracy should be evaluated in the coordinate system of the imaging isocenter. Even though it could be possible indirectly through the laser marker position, that requires multiple steps, which are vulnerable to increase the measurement error. There are alternative methods instead of using films, such as an electronic portal imaging device (EPID),^6^, ^7^, ^8^ ion‐chamber arrays,^9^, ^10^ and scintillation detectors.^11^, ^12^, ^13^, ^14^, ^15^, ^16^ These devices could receive electronic signal and analyze the result in real‐time.

For the radiation isocenter measurement of particle therapy machine, however, above mentioned devices are not suitable. One reason is that particle therapy machine does not support an automated, self‐integrated EPID measurement and analysis system yet. And ion‐chamber arrays have low spatial resolutions, from 5 to 10 mm, which cannot provide sub‐millimeter accuracy. In addition, there is a concern about the radiation damage. Most of the commercial ion‐chamber arrays and scintillation detectors are designed for measuring beam fluence, so the electronics are in the same plane of the device. The radiation beam of star‐lines will pass the electronics and it will be damaged.

In this study, we developed a novel system that aimed to directly compare the radiation isocenter and the imaging isocenter by integrating two measurement systems. We built a seamless QA workflow for measuring and analyzing the radiation/imaging isocenters of proton beams. To validate the performance of our system, we conducted a comparative analysis with the conventional film‐based method. The results of this evaluation demonstrated the efficacy of our system.

## 2 MATERIALS AND METHODS

### 2.1 Measurement device

**FIGURE 1:**
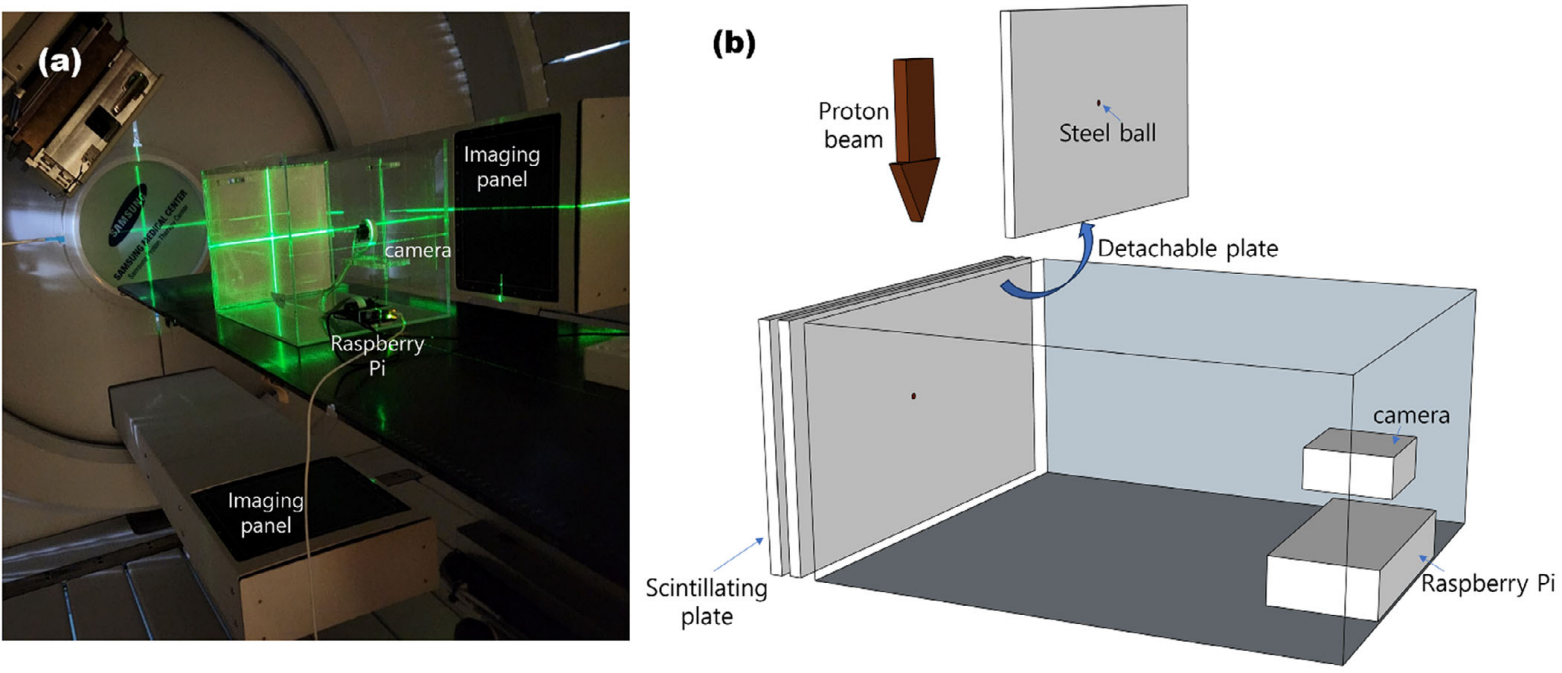
The setup picture of our system on the treatment couch (a) and the schematic diagram of the system (b).

We have developed a radiation isocenter verification system that combines a plastic scintillator (PI‐200, Mitsubishi Chemical Corporation, Tokyo, Japan) whose dimensions is 300 mm × 300 mm × 0.9 mm with a Raspberry Pi 4 system (Raspberry Pi Foundation, London, UK) equipped with a camera module (pi camera V2: a Sony IMX219 8‐megapixel CMOS sensor with a maximum resolution of 3280 × 2464 pixels).^17^ As shown in Figure 1, the scintillator plane was aligned parallel to the direction of radiation for measuring the gantry star‐shot. The camera was installed on an in‐house acrylic phantom to capture the whole scintillator plane. A steel ball with a 2‐mm diameter was attached on a plastic plate to verify the imaging isocenter by using the kilovoltage (kV) X‐ray. And the plate can be easily slid in and out from the phantom. The captured image was transferred to a computer located outside the treatment room connected through an ethernet cable.

Image acquisition from Raspberry Pi was performed by following steps. First, we installed the necessary packages (python, Picamera library) for the camera operation on desktop computer in control room. Then a python script, “stream.py” was scheduled to run automatically when Raspberry Pi was turned on, creating a server that received and streamed camera images. Then using GUI software, we connected desktop computer to Raspberry Pi and controlled the camera.

**FIGURE 2:**
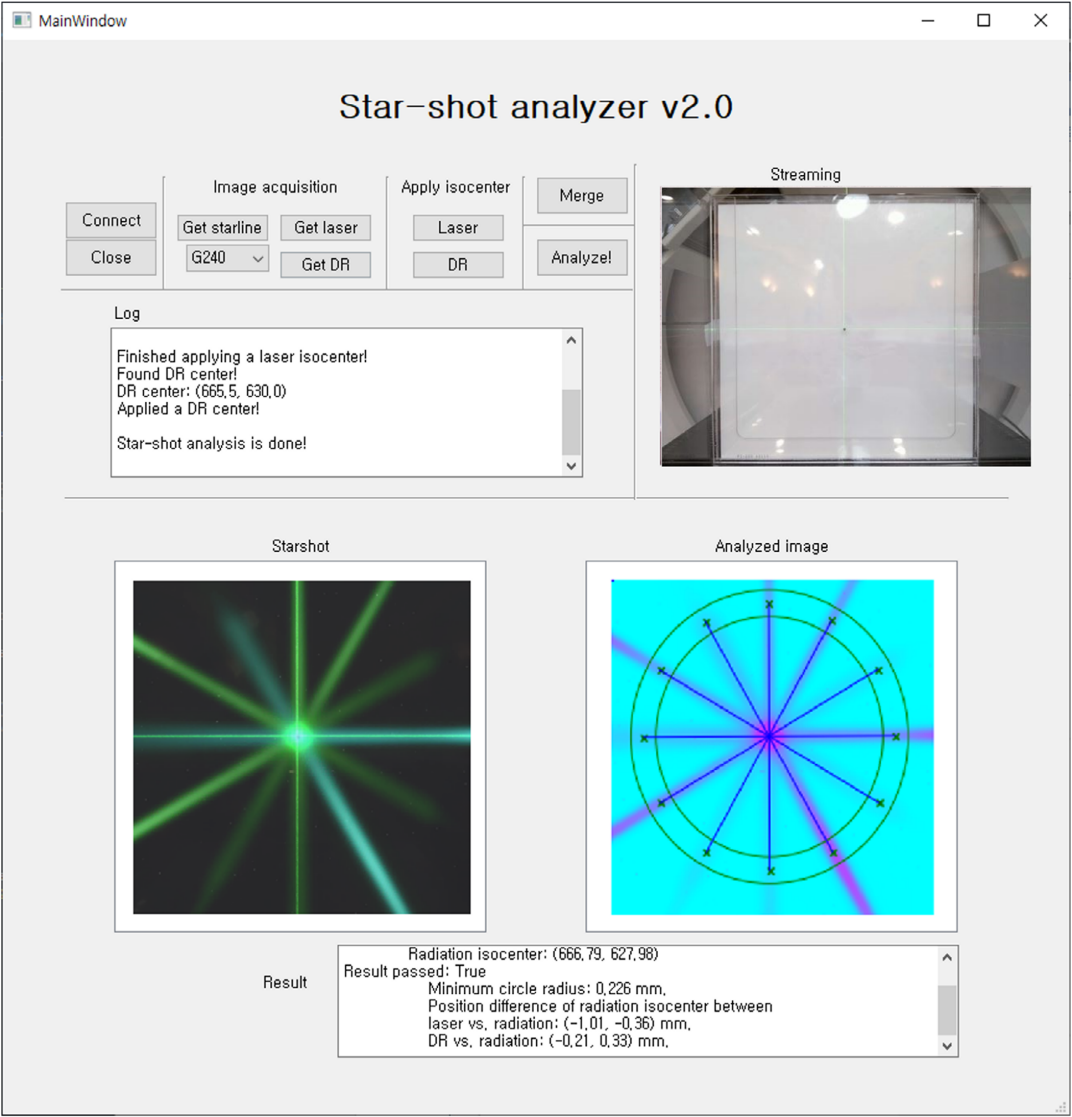
The user interface of in‐house software for analyzing star‐shot image. It displays both the measured star‐shot image and the analysis results. The results include the minimum circle radius and the position difference between the laser (or imaging) and the radiation isocenter.

The user interface of GUI software is presented in Figure 2, displaying both the measured star‐shot image and the analysis results. The analysis results include the minimum circle radius and the position difference between the laser (or imaging) and the radiation isocenter. The source code for GUI software is available online at https://github.com/sagacity98/StarshotAnalyzer.

### 2.2 The star‐shot analysis

The star‐shot analysis of our system follows a four‐step procedure. Initially, the image of a steel ball was captured with the ambient light. Subsequently the plate was removed, a laser image was obtained without the ambient light. Secondly narrow proton beams were delivered, and the scintillating light were captured by the camera. Thirdly, a steel ball and laser images were analyzed to find the pixel location of the imaging isocenter and the laser marker position, respectively. Lastly the system calculated the minimum circle radius and the position difference between the radiation isocenter and the imaging isocenter/laser marker position.

Note that camera calibration is an essential step in rectifying geometric distortions, such as pincushion or barrel distortion of an imaging system.^18^ To achieve this, a checkerboard image is employed as a reference to determine the camera's intrinsic and extrinsic parameters. The result with and without the camera calibration will be compared in the result.

### 2.3 Finding the imaging isocenter

**FIGURE 3:**
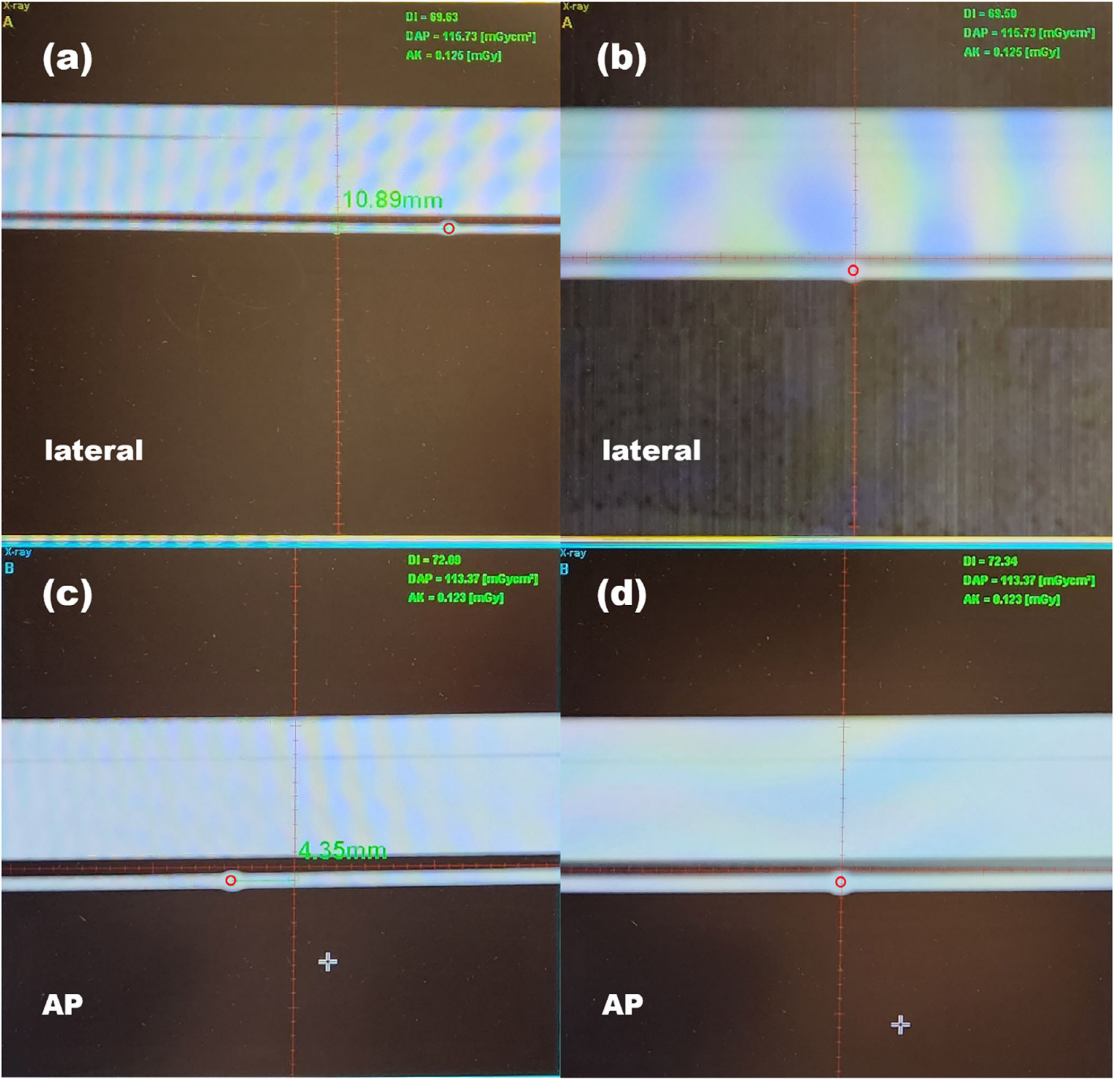
The kV image of the steel ball, before alignment (a, c) and after alignment (b, d). The images were taken at gantry angle 270 degree for (a, b) and 0 degree for (c, d). The red circle represented the position of steel ball in the image and the red line was for the imaging isocenter.

**FIGURE 4:**
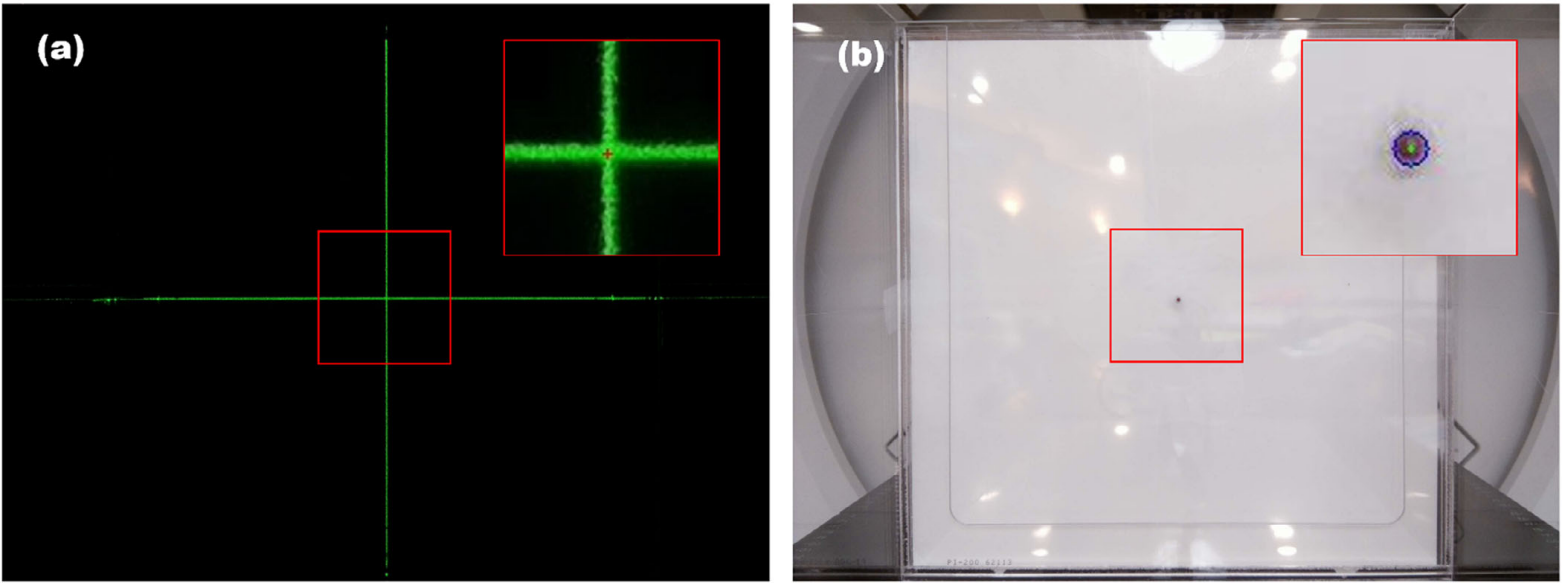
Laser image (green lines) and laser center (red plus) was presented (a). A steel ball image was contoured by blue circle and its center was presented by green dot (b).

A steel ball was positioned near the laser marker position, and kV X‐ray image was obtained by using imaging panel. The imaging system of the treatment machine, MedCom (Darmstadt, Germany), could compute the position difference between the imaging isocenter and the center of the steel ball as shown in Figure 3(a, c). Then we shifted the couch position to align the steel ball at the imaging isocenter, Figure 3(b, d). For the analysis, we took the optical image of the steel ball, and the image was converted into grayscale. The radius of the steel ball was then determined by assessing the size of the contour detected in the image. Finally, the imaging isocenter was determined as the center of the contour as shown in Figure 4(b).

### 2.4 Finding the laser marker position

After aligning the steel ball position to the imaging isocenter, we captured a laser image without any movement, or ambient light. Since the laser width in the image was between 1 and 1.5 mm (equivalent to 8–10 pixels), we had to transform the wide strips of laser light into lines to pinpoint the pixel position of the laser marker. The image was converted to a grayscale image, and we extracted line profiles at different positions, that is, for every 100 pixels interval. By connecting the center positions of the laser strips in the x‐directional line profiles, we obtained a single y‐directional laser line, and it is same for a x‐directional laser line. The pixel position of the intersection point of x and y laser lines was identified as the laser marker position in our system, as depicted in Figure 4(a).

### 2.5 Star‐shot image

We use Sumitomo Heavy Industry (SHI, Japan) proton treatment machine, equipped with a cyclotron capable of delivering energies ranging from 70 to 230 MeV. Gantry 1 featured both wobbling and line‐scanning nozzles, while gantry 2 only had line scanning. Measurements were performed exclusively on gantry 2, delivering proton spot beam with energy 230 MeV and a dose rate 6 MU/s, with 200 MU in service mode.

The star‐shot image was obtained by proton irradiations at various gantry angles, that is, 240°, 300°, 0°, 30°, 90°, and 150°. The proton spot beam was delivered to the origin. And the scintillator converted the proton beam into visible light, which was captured by a camera at each gantry angle, while maintaining a consistent phantom setup. The camera settings were configured as follows: ISO 100, shutter speed 0.09 s, resolution 2726 × 2048 pixels and frame rate 10 frames per second, in the absence of the room light. The camera settings were optimized to minimize the thermal noise on the CCD and prevent the intensity saturation. A single star‐shot image was created by merging every star‐lines from different gantry angles.

### 2.6 Star‐shot analysis

**FIGURE 5:**
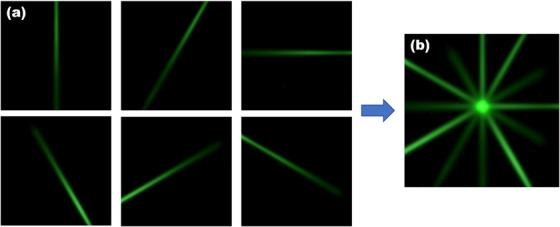
Star‐shot image acquired using a plastic scintillator. Six individual star‐lines were captured by the camera (a). Every star‐line was summed in a star‐shot image (b).

The star‐shot image was analyzed using python library Pylinac to compute the minimum circle radius and the position of the radiation isocenter.^19^ Pylinac calculates the full width at half maximum (FWHM) of each proton beam, transforming them into star‐lines. With six star‐lines, Figure 5, total 15 intersection points were obtained. The minimum circle radius was defined as the radius of the smallest circle encompassing all intersection points from the star‐lines. The position of the radiation isocenter was determined as the center of this minimum circle.

### 2.7 Comparison with EBT3 film

We compared the results of star‐shot analysis obtained from our system and Gafchromic EBT3 film. The EBT3 film was attached behind the scintillator, the laser position was marked on the film, and proton beams were delivered to both our system and EBT3 film simultaneously. After that, the films were scanned with 300 dots per inch (dpi) and the results were analyzed using Pylinac.

## 3 RESULTS

We obtained five star‐shot images using both EBT3 film and our system. Each measurement was made on different days to evaluate the effect of random setup error. The minimum circle radii are presented in Table 1. The average minimum circle radius were 0.41 ± 0.10 mm for EBT3 film and 0.29 ± 0.14 mm with our system. The average position differences between the radiation isocenter and the laser marker position were (−0.47 mm, −0.97 mm) for EBT3 film, (−0.89 mm, −0.39 mm) for our system, with the deviations in the x and y directions denoted in parentheses as (x, y). The average value of position difference between the radiation isocenter and the imaging isocenter was (−0.23 mm, 0.14 mm) in our system. We applied a camera calibration factor to the images, but the difference between the calibrated and non‐calibrated images was negligible—less than ±0.07 mm, as shown in Table 1. Therefore, non‐calibrated images were used for this work.

**TABLE 1:** The result of isocenter verification using EBT3 film and our system without applying camera calibration.

Day			The position difference in (x, y) of radiation isocenter (mm) to		
	Minimum circle radius (mm)		EBT3 film	Our system	
	EBT3 film	Our system	Laser marker position	Laser marker position	Imaging isocenter
1	0.57	0.21 0.20^*^	(−0.65, −0.85)	(−0.83, −0.42) (−0.90, −0.43)^*^	(−0.36, 0.27) (−0.43, 0.25)^*^
2	0.42	0.55 0.54^*^	(−0.23, −1.11)	(−0.77, −0.61) (−0.81, −0.61)^*^	(0.11, 0.11) (0.07, 0.15)^*^
3	0.40	0.16 0.12^*^	(−0.65, −0.95)	(−0.97, −0.44) (−1.01, −0.38)^*^	(−0.16, −0.12) (−0.22, −0.05)^*^
4	0.41	0.28 0.24^*^	(−0.65, −0.98)	(−0.87, −0.17) (−0.91, −0.10)^*^	(−0.52, 0.13) (−0.54, 0.15)^*^
5	0.26	0.23 0.20^*^	(−0.17, −0.95)	(−1.09, −0.32) (−1.11, −0.28)^*^	(−0.21, 0.33) (−0.21, 0.36)^*^

*Note*: Asterisk is the result with camera calibration.

**FIGURE 6:**
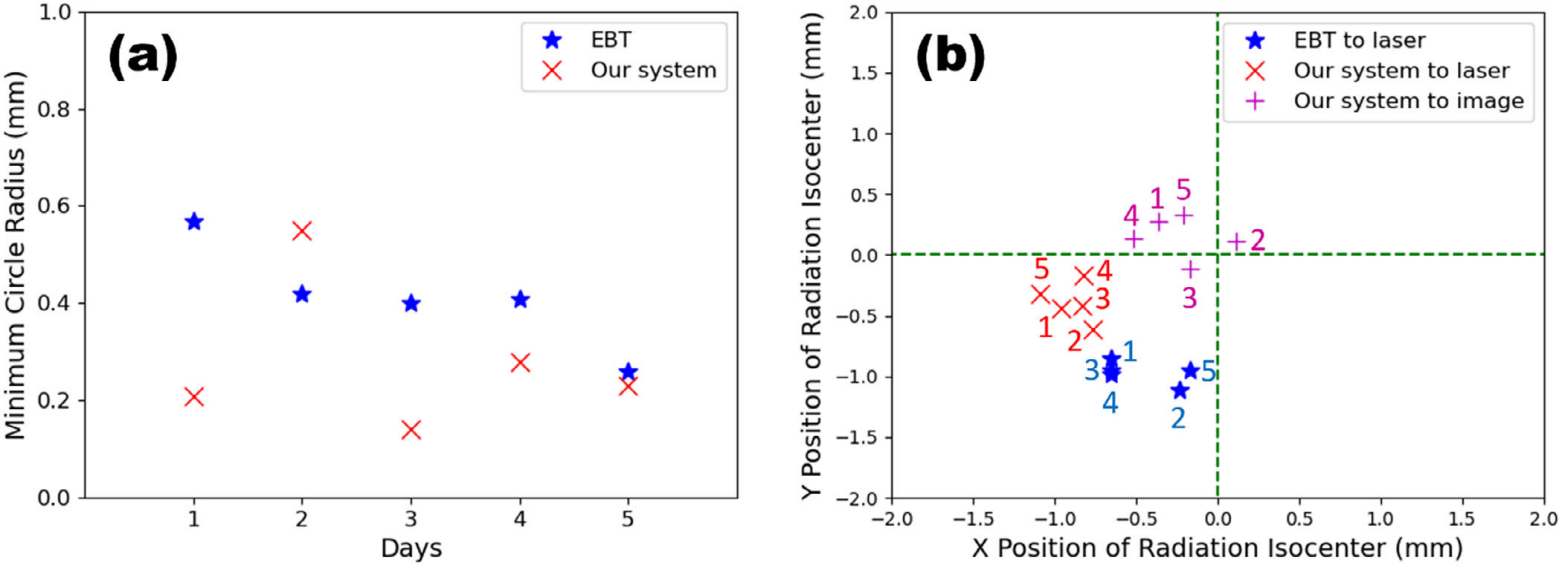
The minimum circle radius (a) and the position difference between the laser (or imaging) and the radiation isocenter (b) are plotted for EBT film (blue star) and our system (red cross for the laser, magenta plus for the imaging). The numbers in the figure (b) represent the day of measurements.

All five measurements obtained by our system had minimum circle radii less than 0.55 mm as shown in Figure 6(a). The relative positions of the radiation isocenter from the laser marker position by our system (red cross), imaging isocenter by our system (magenta plus), and laser marker position by the EBT3 film (blue star) are presented in Figure 6(b). The maximum deviations of the radiation isocenter position are as follows: 1.18 mm for the laser marker position by EBT3 film, 1.14 mm for the laser marker position by our system, and 0.54 mm for the imaging isocenter by our system.

## 4 DISCUSSIONS

We have developed a system which can compute the difference between radiation isocenter and imaging isocenter directly. The system used an optical camera to take images of a steel ball, laser marker and the star‐shot image. Since all images were obtained in a single framework, we could compare the difference directly. The results obtained by our system were in good agreement with the results by EBT3 film, and suitable to use for verifying the radiation isocenter position according to AAPM Reports 142^1^ and 224.^2^ It demonstrates that the performance of our system is comparable to the conventional film‐based method.

On the other hand, comparing indirectly through the laser marker position requires multiple steps. To address this issue, many researchers have attempted to integrate multiple isocenter measurements into one system or procedure.^20^, ^21^, ^22^, ^23^ Note that Fuse et al. developed a new device which can compute the offset value of the radiation isocenter from the imaging isocenter by using the radiosensitive film.^24^ And modified Winston Lutz test could compare radiation isocenter to mechanical isocenter,^25^ to imaging isocenter.^26^ However, the analysis cannot be done in real time since they used radiosensitive films. There are a lot of research on using 2D scintillating screen for radiotherapy QA.^11^, ^12^ Most of these studies are designed to use patient‐specific QA^13^ or spot size measurement for particle therapy.^14^, ^15^ Commercial products are also available, Lynx (IBA Dosimetry, Schwarzenbruck, Germany)^27^ and XRV‐124 (Logos Vision System, CA, USA).^28^ Especially, XRV‐124 computes the offset value of the radiation isocenter from the imaging isocenter in real time.^29^, ^30^ But the purchasing price is much higher than our system.

Our system provides cost reductions in both time and money. Conventional film‐based measurement involves multiple procedures, such as marking the laser position on the film, scanning the film, and transferring data to a computer for the analysis. These multiple steps are performed in different locations, for instance, marking is done in the treatment room while scanning is done in another place where a scanner is installed. In contrast, our system enables all these processes to be carried out in a single location, the treatment room, resulting in a significant reduction of total time. The total time spent by film‐based measurement and analysis is 45 min, and by our system is 35 min for a single gantry.

Another notable advantage of our system lies in its cost‐effectiveness. In a film‐based method, the total cost can accumulate due to the need for multiple measurements, each costing tens of US dollars. In contrast, our system is reusable, and its manufacturing cost is reasonable. The system consists of Raspberry pi, open‐source libraries, and in‐house acrylic phantom, the cost is, approximately 200 US dollars. And the scintillator is about 2000 US dollars. By comparing two methods, in 1 year, our system takes 2200 US dollars, but film‐based measurement takes 600 US dollars for one gantry if star‐shot measurement was done monthly. If two gantries are used, the cost for the film will be 1200 US dollars. Notably, our system will not charge additional cost whether how many gantries are used or how many measurements are done until the system breaks down. When considering the total cost, our system proves to be a more economical than the film‐based method.

Our imaging system, a CCD camera, was sensitive to the secondary particles including neutrons generated from the nozzle. Passive scattering beams produce more secondary particles than scanning beams,^31^ resulting in greater damage to the CCD camera.^32^ When we measured passive scattering beams, the CCD camera captured significant noise from the secondary particles. Adding shielding materials to the acrylic phantom could protect the camera, but it increases the size and the weight of the system. Therefore, we determined not to shield the phantom and to only use our system for scanning beam measurements. We tested our system about a year with same Raspberry Pi and camera module, there is no electronic malfunctions. If any component failed, we could replace them easily, the price is less than 100 US dollars. On the other hand, the plastic scintillator would be damaged by aging^33^ and repeated radiation exposure.^34^ It resulted the wavelength shifting and the intensity degradation of the scintillating light. However, this degradation did not significantly impact the star‐shot analysis.

Another shortage of our system is that it cannot specify the 3‐dimensional position of radiation isocenter, unlike Winston‐Lutz test. We can only measure the radiation isocenter position on the star‐shot plane which is parallel to the beam. In other words, we cannot determine the isocenter position in target‐gun direction. Film‐based star‐shot analysis has the same limitation. If we measure the position of proton spot beam irradiated in a plane perpendicular to the beam, we can determine the left‐right and target‐gun position of radiation isocenter. But it still cannot determine the isocenter position in anterior‐posterior direction. To measure the three‐dimensional position of radiation isocenter position, we need to develop another method and it is beyond our work.

The average minimum circle radius of our system was 0.29 mm, which is smaller than EBT3 film measurement of 0.41 mm. In terms of the pixel resolution, the minimum circle radii were 1.74 pixels for our system and 4.85 pixels for EBT3 film. The vector magnitudes showed an isocenter difference of 0.91 mm (5.66 pixels) with our system (laser vs. radiation), 0.35 mm (2.16 pixels) with our system (imaging vs. radiation), and 1.10 mm (12.98 pixels) with EBT3 film (laser vs. radiation). We scanned the EBT3 film at 300 dpi, whereas our system captured the image at a resolution of 2726 × 2048 pixels. The resolution of the EBT3 film is 0.08 mm/pixel, our system has a resolution of 0.16 mm/pixel, which is about twice as poor. This means our system gives the equivalent result with EBT3 film even using a lower image resolution.

## 5 CONCLUSIONS

In this study, we developed and evaluated a cost‐effective scintillation system for measuring the star‐shot of proton beam. Our system demonstrated rapid and precise analysis capabilities with results showing reproducibility and comparability to the conventional film‐based method. Our system allows for quantitative analysis of the star‐shot image of the proton beam, including the minimum circle radius and the position difference between the radiation isocenter and the imaging center. To improve usability, we created an in‐house software using open‐source Python libraries, available on GitHub.

## AUTHOR CONTRIBUTIONS

Ji Hye Han created python GUI code and measured/analysed the data. Kwanghyun Jo conceived the present work and drafted the manuscript primarily. And all authors provided feedback before the final draft submission.

## CONFLICT OF INTEREST STATEMENT

There is no conflict of interest.
